# Stress Markers, Executive Functioning, and Resilience Among Early Adolescents With Complex Congenital Heart Disease

**DOI:** 10.1001/jamanetworkopen.2023.55373

**Published:** 2024-02-09

**Authors:** Lilian von Werdt, Tina M. Binz, Ruth Tuura O’Gorman, Alenka Schmid, Nadja Naef, Valentin Rousson, Oliver Kretschmar, Rabia Liamlahi, Bea Latal, Melanie Ehrler

**Affiliations:** 1 Child Development Center, University Children’s Hospital Zurich, Switzerland.; 2 Children’s Research Centre, University Children’s Hospital Zurich, Switzerland.; 3 Center for Forensic Hair Analytics, Zurich Institute of Forensic Medicine, University of Zurich, Switzerland.; 4 Center for MR Research, University Children’s Hospital Zurich, Switzerland; 5 Division of Biostatistics, Center for Primary Care and Public Health (Unisanté), University of Lausanne, Switzerland; 6 Pediatric Cardiology, Pediatric Heart Center, Department of Surgery, University Children’s Hospital Zurich, Switzerland.; 7 University Research Priority Program (URPP), Adaptive Brain Circuits in Development and Learning (AdaBD), University of Zurich, Switzerland

## Abstract

**Importance:**

Infants with complex congenital heart disease (cCHD) may experience prolonged and severe stress when undergoing open heart surgery. However, little is known about long-term stress and its role in neurodevelopmental impairments in this population.

**Objective:**

To investigate potential differences between early adolescents aged 10 to 15 years with cCHD and healthy controls in physiological stress markers by hair analysis, executive function (EF) performance, and resilience.

**Design, Setting, and Participants:**

This single-center, population-based case-control study was conducted at the University Children’s Hospital Zurich, Switzerland. Patients with different types of cCHD who underwent cardiopulmonary bypass surgery during the first year of life and who did not have a genetic disorder were included in a prospective cohort study between 2004 and 2012. A total of 178 patients were eligible for assessment at ages 10 to 15 years. A control group of healthy term-born individuals was cross-sectionally recruited. Data assessment was between 2019 and 2021. Statistical analysis was performed from January to April 2023.

**Exposure:**

Patients with cCHD who underwent infant open heart surgery.

**Main Outcomes and Measures:**

Physiological stress markers were quantified by summing cortisol and cortisone concentrations measured with liquid chromatography with tandem mass spectrometry in a 3-centimeter hair strand. EFs were assessed with a neuropsychological test battery to produce an age-adjusted EF summary score. Resilience was assessed with a standardized self-report questionnaire.

**Results:**

The study included 100 patients with cCHD and 104 controls between 10 and 15 years of age (mean [SD] age, 13.3 [1.3] years); 110 (53.9%) were male and 94 (46.1%) were female. When adjusting for age, sex, and parental education, patients had significantly higher sums of hair cortisol and cortisone concentrations (β, 0.28 [95% CI, 0.12 to 0.43]; *P* < .001) and lower EF scores (β, −0.36 [95% CI, −0.49 to −0.23]; *P* < .001) than controls. There was no group difference in self-reported resilience (β, −0.04 [95% CI, −0.23 to 0.12]; *P* = .63). A significant interaction effect between stress markers and EFs was found, indicating a stronger negative association in patients than controls (β, −0.65 [95% CI, −1.15 to −0.15]; *P* = .01). The contrast effects were not significant in patients (β, −0.21 [95% CI, −0.43 to −0.00]; *P* = .06) and controls (β, 0.09 [95% CI, −0.11 to 0.30]; *P* = .38).

**Conclusions and Relevance:**

This case-control study provides evidence for altered physiological stress levels in adolescents with cCHD and an association with poorer EF. These results suggest that future studies are needed to better understand the neurobiological mechanisms and timing of alterations in the stress system and its role in neurodevelopment.

## Key Points

**Question:**

Do early adolescents with complex congenital heart disease (cCHD) have higher stress markers than healthy controls, and is there an association with executive functions and resilience?

**Findings:**

In this case-control study of 100 early adolescents with cCHD and 104 controls, those with cCHD had higher stress markers in hair and lower executive function scores than those without cCHD. A significant interaction effect was observed between stress markers and executive function.

**Meaning:**

This study’s results suggest evidence for clinically relevant alteration in physiological stress markers in adolescents with cCHD and an association with neurodevelopmental sequelae.

## Teaser

This case-control study compares physiological stress markers, executive function, and resilience among individuals aged 10 to 15 years with complex congenital heart disease vs healthy controls.

## Introduction

Infants with complex congenital heart disease (cCHD) often undergo multiple surgical procedures, intensive care unit (ICU) stays, and medical interventions. This likely causes intense and prolonged stress.^1^ Infant stress can disturb the hypothalamic-pituitary-adrenal (HPA) axis and the release of cortisol, which regulates normal stress reactions.^2,3,4^ Studies have investigated the association of early life stress and cortisol levels in infants with cCHD. Altered serum and salivary cortisol levels were associated with more complicated ICU stays and differed from reference values in children undergoing open heart surgery preoperatively and postoperatively.^5,6^ Another study found that cortisol levels differed between children aged 3 to 5 years who had undergone infant cardiac surgery and children with cCHD who did not require surgery.^7^ Whereas salivary and serum cortisol reflects acute stress, hair cortisol reflects a longer-term concentration,^8^ which constitutes a biomarker for chronic stress and persistent alterations in the HPA axis.^9^ This technique has proved successful in other studies investigating children and adolescents^10^ but has not been applied in studies involving patients with cCHD. Furthermore, the clinical relevance of altered stress markers in patients with cCHD remains unclear.

Children and adolescents with cCHD are at risk for executive function (EF) impairments.^11,12^ EFs are higher-order cognitive functions relevant for goal-directed behavior and to regulate cognition, behavior, emotions, and social functioning.^13^ However, the mechanisms underlying poor EFs in patients with cCHD remain unclear. An exploratory study in 15 children with tetralogy of Fallot found that the morphology of the inferior parietal gyrus was associated with preoperative cortisone levels and cognition. However, a direct link between cortisone/cortisol and cognition was not examined.^14^ Overall, little is known about the influence of stress on EFs in cCHD, but studies in healthy individuals indicate a link between low EF performance and high stress measured by cortisol levels.^15,16,17^ The underlying mechanisms remain unclear. However, cortisol can cross the blood-brain barrier and may be harmful for brain structure and function.^18^ Brain structures relevant to EFs (eg, prefrontal cortex, hippocampus) are particularly vulnerable to increased cortisol in humans and animal models.^19,20^ In contrast, EF difficulties may compromise psychological well-being due to increased academic and socioemotional difficulties, which in turn can increase stress.

While stress and high cortisol levels may be risk factors for lower EF and vice versa, better EF performance has been shown to correlate with better resilience in healthy children, adolescents,^21^ and young adults.^22^ “Resilience is a process that allows an individual to adapt to difficult life experiences requiring mental, emotional, and behavioral flexibility”^23^ and can be assessed with standardized questionnaires.^24^ To date, research investigating resilience in patients with cCHD is limited. However, a recent study showed adolescent patients with cCHD score lower than controls in a resilience questionnaire.^25^ Interindividual differences in resilience may mitigate both stress and EF impairments in the cCHD population, but research evidence is lacking.

We aimed to investigate if patients with cCHD had higher stress markers, quantified by hair cortisol and cortisone levels, lower EF performance, and lower resilience than healthy controls. Furthermore, we aimed to test the correlations between stress markers, EFs, and resilience.

## Methods

### Study Design and Population

This case-control study is part of a prospective cohort study (Teen Heart Study^26^*)* investigating neurodevelopmental outcomes and cerebral magnetic resonance imaging in early adolescents with cCHD and was conducted at the University Children’s Hospital Zurich, Switzerland, between April 2019 and September 2021.

This study included patients with cCHD who underwent cardiopulmonary bypass surgery (CPB) between 2004 and 2012 at the University Children’s Hospital Zurich. Patients were eligible if they underwent CPB surgery before 1 year of age, were not diagnosed with a genetic or dysmorphic syndrome, and were between 10 and 15 years of age at the time of assessment. The age range of 10 to 15 years has previously been defined as early adolescence.^27^ Of 178 eligible patients, 100 early adolescents with cCHD participated in the current study (participation rate: 56%). A control group of 104 healthy early adolescents between 10 and 15 years of age was recruited with the following exclusion criteria: preterm birth (<36 weeks of gestation), diagnosed with a neurological or substantial developmental disorder (eg, learning disorder or attention deficit hyperactivity disorder). Controls were recruited as friends of participating patients, through print and online advertisement. See the eFigure in Supplement 1 for the recruitment procedure. The study was approved by the ethics committee of the Canton of Zurich. Written informed consent was obtained prior to participation from the participants’ legal guardians and from participants if they were 14 years and older. The Strengthening the Reporting of Observational Studies in Epidemiology (STROBE) reporting guideline was followed.

### Measures

#### Stress Markers

The analysis of the stress markers, cortisol and its metabolite cortisone, was conducted using 1 strand of hair collected from the posterior vertex region. A 3-centimeter hair segment was cut proximally to the scalp. At a mean growth rate of 1-centimeter per month, this represents the cumulative cortisol and cortisone concentration of the past 3 months. The sample was stored in aluminum foil at room temperature. Cortisol and cortisone were analyzed and quantified by liquid chromatography coupled with tandem mass spectrometry following a protocol by Voegel and colleagues.^28^ The sum of cortisol and cortisone was calculated for further statistical analysis as suggested by Voegel and colleagues.^29^

#### Executive Functions

EFs were investigated with an extensive standardized neuropsychological test battery.^26,30,31,32,33,34^ Details about the neuropsychological test measures of EFs are displayed in Table 1. A summary score for overall EF performance was built from these tests. See eText 1 and eTables 1, 2, and 3 in Supplement 1 for details.

**Table 1.** Neuropsychological Test Battery to Assess Executive Functions Performance

Executive function domains	Neuropsychological test	Test measurement
Working memory	Digit span forward and backward (WISC-IV)	No. of correct items
	Letter-number sequencing (WISC-IV)	No. of correct items
	Corsi block tapping test (Corsi)	No. of correct items
Inhibition	Subtest interference, color word interference task (D-KEFS)	Completion time
	Go/NoGo (TAP)	No. of commission errors
Cognitive flexibility	Subtest letter-number-switching, trail making task (D-KEFS)	Completion time
	TAP flexibility (TAP)	Median reaction time
Fluency	Subtests s-words and animals (RWT)	No. of correct items
	Subtest filled-dots-only, design fluency test (D-KEFS)	No. of correct items
Planning	Tower task (D-KEFS)	Total achievement score

Abbreviations: D-KEFS, Delis-Kaplan Executive Function System; TAP, Test of Attentional Performance; RWT, Regensburger Verbal Fluency Test; WISC-IV, Wechsler Intelligence Scale for Children Fourth Edition, Corsi.

#### Resilience

Resilience was assessed with the Resilience Scale 13 (RS-13), which measures self-reported personality traits of acceptance of self and life and personal competences and has been validated in a German-speaking population. This questionnaire has good internal consistency and acceptable test-retest reliability, can be used in adolescents and adults, and has been applied in various patient samples.^35,36^ Participants rate the accuracy of 13 statements on a 7-point Likert scale. Higher scores indicate higher resilience. The sum score was calculated and used for statistical analysis.^35^

#### Participant Characteristics

Medical information was collected prospectively from patients’ medical records and included information on the neonatal period, the cCHD physiology, and the perioperative period. Sociodemographic variables were assessed in a parent-reported questionnaire: Parental education was calculated by the sum of maternal and paternal highest education, each on a 6-point scale (from 1 = no high school qualification to 6 = university degree).^37^ A 6-point Likert scale assessed whether their financial resources were sufficient to cover their living costs. Parental immigration background was assessed. Race and ethnicity were not assessed as part of this study as these variables were not included in the demographic questionnaire.

IQ was evaluated with a corrected short version of the Wechsler Intelligence Scale for Children 4th edition, including the subtests of matrices, similarities, letter number sequencing, and symbol search.^38,39^ The parent-reported life events scale assessed whether and/or which of 13 potentially stressful events (eg, parental separation, illness/death of a relative or familiar person) happened within the past 3 months.^40^ A sum score from 0 to 13 was calculated.

### Statistical Analysis

For hypotheses testing, linear regression analysis was used to assess group differences (patients with cCHD vs controls) with respect to stress markers, EFs, and resilience, as well to investigate the association among these 3 outcomes. Post hoc, we investigated differences between patients with a univentricular and a biventricular cCHD regarding stress markers, EFs, and resilience. Furthermore, we tested an association between stress markers and clinical variables. At last, an interaction effect (group × cortisol) for the outcome EFs was tested and contrast effects per group were investigated post hoc. All post hoc analyses were false discovery rate–corrected. For consistency, all analyses were adjusted for sex, age, and parental education. Parental education was missing in 12 patients and 12 controls (12%) and was imputed by chained equation with 1 imputation and 5 iterations.^41^ All outcomes and factors included in the regression models were used for estimation of missing values. Standardized β and unstandardized B coefficients with 95% CIs were reported. Models were visually investigated for normal distribution of residuals.

To help interpret the EF summary score, each EF test contributing to the summary score was compared between patients and controls with a 2-sampled *t* test and between patients and available normative data with a 1-sampled *t* test. Results were considered statistically significant if 2-sided *P* < .05. All analyses were conducted using R version 4.2.0 (R Project for Statistical Computing) from January to April 2023.^42^

## Results

### Population Characteristics

The study included 100 patients with cCHD and 104 controls between 10 and 15 years of age; mean [SD] age was 13.3 [1.3] years; 110 (53.9%) were male and 94 (46.1%) were female; on average they were term-born with a mean (SD) gestational age of 39.2 (1.8) weeks. Demographic and clinical characteristics stratified by group are reported in Table 2. Statistical estimates of all EF tests that contributed to the EF summary score are displayed in eTable 4 in Supplement 1. Three cortisol-plus-cortisone samples were excluded due to suspected contamination. Patients who participated in this study did not significantly differ from patients who were eligible but rejected participation in clinical and demographic variables (eText 2 in Supplement 1).

**Table 2.** Demographic and Clinical Characteristics Stratified by Group

Population characteristics	cCHD (n = 100)	Control (n = 104)	*P* value
Innate			
Sex, No. (%)			
Female	39 (39.0)	55 (52.9)	.07^a^
Male	61 (61.0)	49 (47.1)	.07^a^
Age, mean (SD), y	13.7 (1.2)	13.0 (1.4)	<.001^b^
Parental education, median (IQR)	8 (6-9)	10 (8-12)	<.001^b^
Parental immigration background, No. (%)	20 (20.0)	13 (12.5)	.21^a^
Sufficient financial resources, median (IQR)	6 (5-6)	6 (5-6)	.003
Life events in past 3 mo, median (IQR)	0 (0-1)	0 (0-1)	.17
Gestational age, mean (SD), wk	39.1 (2.0)	39.2 (1.5)	.65^b^
IQ, mean (SD)	99.2 (14.4)	110.8 (9.2)	<.001^b^
Prenatal diagnosis, No. (%)	23 (23.0)	NA	NA
Cyanotic cCHD, No. (%)	73 (73.0)	NA	NA
Biventricular cCHD, No. (%)	76 (76.0)	NA	NA
Preoperative (1st CPB surgery)			
Preoperative saturation, mean (SD)	86.4 (10.1)	NA	NA
Age at surgery, mean (SD), mo	2.4 (2.8)	NA	NA
Intraoperative (1st CPB surgery)			
Lowest temperature, mean (SD), °C	29.0 (4.2)	NA	NA
Time on ECC, mean (SD), min	169.5 (71.3)	NA	NA
Postoperative (1st CPB surgery)			
Time in ICU, median (IQR), d	8 (5-13)	NA	NA
Time in hospital, median (IQR), d	26.5 (18.0-39.2)	NA	NA
No. CPB surgical procedures, median (IQR)	1 (1-2)	NA	NA

Abbreviations: cCHD, complex congenital heart disease; CPB, cardiopulmonary bypass; ECC, extracorporeal circulation; ICU, intensive care unit; NA, not applicable (these data do not exist because the controls did not undergo surgery and were not hospitalized).

^a^ Two-sampled χ^2^ test.

^b^ Two-sampled; two-sided Mann-Whitney *U* test. OR for χ^2^ tests; Cohen *d* for *t* tests; *r* for Mann-Whitney *U* test.

### Group Comparisons

**Figure 1.**
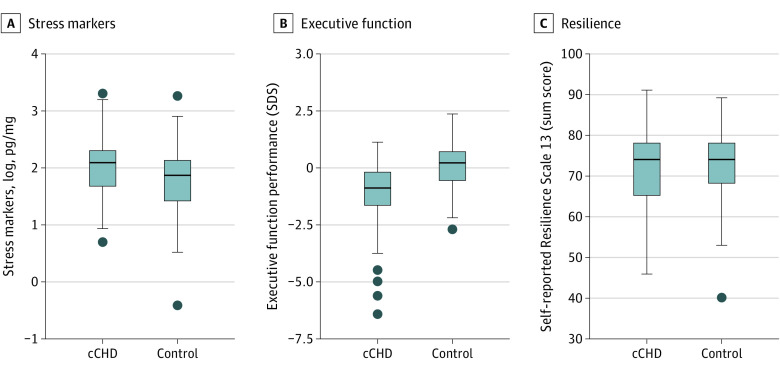
Group Comparisons for Stress Markers, Executive Functions, and Resilience The lower and upper borders of the box represent the first and third quartiles. The line within the box corresponds to the median. Outliers are represented by circles. cCHD indicates complex congenital heart disease; SDS, standard deviation score.

Data on stress markers was skewed and was thus transformed with the natural logarithm to reach normality of data distribution. Compared with controls, patients had significantly higher stress makers quantified by mean (SD) cortisol-plus-cortisone concentrations (cCHD: 9.1 [5.4] pg/mg; controls: 6.8 [3.8] pg/mg; cCHD log-transformed: 2.05 [0.56]; controls log-transformed: 1.76 [0.59]; β, 0.28 [95% CI, 0.12 to 0.43]; *P* < .001) and lower mean (SD) EFs (CHD: −1.2 [1.4]; controls: 0.01 [1.0]; β, −0.36 [95% CI, −0.49 to −0.23]; *P* < .001). There was no significant difference in mean (SD) resilience sum scores between patients and controls (CHD: 71.7 [9.4]); controls: 73.4 [8.5]; β, −0.04 [95% CI, −0.23 to 0.12]; *P* = .63) (Figure 1 and Table 3). Female early adolescents had higher levels of cortisol plus cortisone and higher scores for resilience than male early adolescents.

**Table 3.** Linear Regression Models Estimating Group Differences^a^^,^^b^

Outcome	Factors	β (95% CI)		*P* value	*R*^2^ adjusted	*P* value model fit
		Standardized	Unstandardized			
Stress markers	Group	0.28 (0.12 to 0.43)	0.33 (0.14 to 0.52)	<.001	0.10	<.001
	Sex	0.25 (0.11 to 0.39)	0.3 (0.12 to 0.47)	<.001		
	Age	−0.03 (−0.18 to 0.11)	−0.01 (−0.08 to 0.05)	.66		
	Parental education	−0.01 (−0.16 to 0.14)	−0.002 (−0.04 to 0.04)	.90		
Executive functions	Group	−0.36 (−0.49 to −0.23)	−0.93 (−1.30 to −0.57)	<.001	0.23	<.001
	Sex	0.01 (−0.14 to 0.11)	0.03 (−0.36 to 0.30)	.84		
	Age	0.02 (−0.1 to 0.15)	0.02 (−0.10 to 0.15)	.71		
	Parental education	0.24 (0.11 to 0.37)	0.12 (0.05 to 0.20)	.001		
Resilience	Group	−0.04 (−0.23 to 0.12)	−0.72 (−3.67 to 2.23)	.63	0.09	.07
	Sex	0.02 (0.05 to 0.34)	3.55 (0.85 to 6.24)	.01		
	Age	−0.05 (−0.2 to 0.1)	−0.36 (−1.40 to 0.69)	.50		
	Parental education	0.05 (−0.11 to 0.21)	−0.18 (−0.40 to 0.76)	.54		

^a^ Reference for group comparison: controls. Reference for sex comparison: male individuals.

^b^ Missing data: stress marker (n = 19 [9%]), executive function summary score (n = 6 [3%]), Resilience Scale 13 sum score (n = 30 [15%]).

There was no significant difference in stress markers between participants who experienced 1 or more life events the past 3 months compared with those who did not (*t* = –0.75; *P* = .46; ≥1 life events: 40 of 100 patients [40.0%], 31 of 104 controls [29.8%]). Post hoc analysis found no significant difference between patients with univentricular and biventricular cCHD in stress markers, EFs or resilience (eTable 5 in Supplement 1). Lastly, post hoc analysis showed no significant association between stress markers and any clinical variables (eTable 6 in Supplement 1).

### Associations Between Stress Markers, EFs, and Resilience

**Figure 2.**
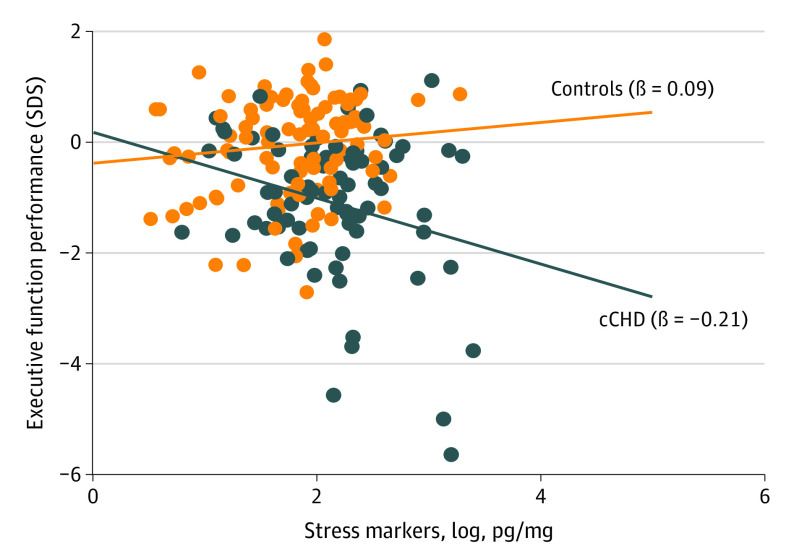
Association of Executive Function and Stress Markers Stratified by Group Individual data points are represented by dots. Lines represent linear regressions for patients and controls. SDS indicates standard deviation score.

Stress markers were not significantly associated with EFs over the whole sample, but there was a significant interaction, indicating that the association between EFs and stress markers depended on group (β, −0.65 [95% CI, −1.15 to −0.15]; *P* = .01). Post hoc, there was a significant interaction, indicating that the association between EFs and stress markers depended on group. The association between higher stress markers and lower EFs was stronger in patients than in controls (ie, interaction), but these contrast effects were not significant (patients: β, −0.21 [95% CI, −0.43 to −0.00]; *P* = .06; controls: β, 0.09 [95% CI, −0.11 to 0.30]; *P* = .38) (Figure 2; eTable 7 in Supplement 1). Resilience was not significantly associated with stress markers or EFs (eTable 8 in Supplement 1).

## Discussion

To our knowledge, this is the first study that has investigated physiological stress markers and their associations with EF impairments in a large sample of early adolescents with cCHD. We found that early adolescents with cCHD (aged 10 to 15 years) have elevated long-term hair cortisol-plus-cortisone concentration. Furthermore, patients with cCHD had significantly lower EF performance than controls, but there was no difference in self-reported resilience. We identified a significant interaction effect, indicating that a negative association between stress markers and EFs is stronger in patients than in controls.

### Altered Stress Markers in Early Adolescents With cCHD

Several studies have suggested that high early life stress may alter the function of the HPA axis and consequently alter stress markers such as cortisol and cortisone.^43,44^ Indeed, studies in infants and young children with cCHD have demonstrated that altered stress markers, measured by serum and salivary cortisol, are associated with complications during the ICU stay such as prolonged mechanical ventilation and inotrope medication and with the cardiopulmonary bypass surgery.^5,6,7^

In our cohort, we found no significant association between stress markers in hair at ages 10 to 15 years and length of ICU stay following open heart surgery in infancy and other clinical variables. However, length of ICU stay may not be a sufficiently sensitive marker for early life stress during hospitalization. We further found no difference in stress markers between patients with univentricular and biventricular cCHD, although patients with univentricular cCHD may be expected to experience more early life stress than those with biventricular cCHD, because univentricular patients typically undergo repeated open heart surgery during infancy, with prolonged hospital and ICU stays entailing higher risk of complications. Stressful events experienced during early life by patients with cCHD can be manifold and include physical pain (eg, pin picks due to needle injections), intubation, noise on the ICU, and emotional stress (eg, due to separation from parents). Future studies should examine such events separately and investigate associations with stress markers.

Although we did not find differences between subgroups of patients with cCHD, we found evidence for increased stress markers in the whole cCHD group. Early life stress may be one explanation for increased cortisol-plus-cortisone concentration during adolescence. Other explanations for higher stress markers in early adolescents with cCHD could be continued high stress associated with higher rate of emotional problems,^45^ and neurodevelopmental impairments at the time of measurement.^46,47^ Longitudinal studies are needed to assess perceived stress and biological stress markers starting in early life.

### Stress Markers and Executive Function Performance

Previous meta-analyses have suggested that both early life and acute stress are associated with lower EF performance.^48,49^ Studies have reported mixed findings on an association between cortisol levels, EF, and overall cognitive performance in healthy individuals.^50,51,52,53,54^

Nits and colleagues^55^ proposed the neonatal stress embedding (NSE) model in the preterm population, which provides a comprehensive description of the biological embedding of stress and how this may affect long-term neurodevelopmental outcomes. The NSE model suggests that the effect of early life stress on neurodevelopment may be mediated by changes in the immune system, the autonomic nervous system, the HPA axis, and gene expressions, and that this effect may be moderated by the prenatal environment and parent–infant interactions. (Refer to Nits et al^55^ for details.)

In the current study, we observed higher levels of stress markers and lower EF performance in patients than controls and a significant group interaction for the association between stress markers and EF. Post hoc analysis showed that the association between higher stress markers and lower EF was higher in patients than in controls. These effects were small and did not survive multiple comparison correction. Thus, future studies should replicate these findings in a well-powered sample. However, these findings show evidence that alterations of the physiological stress system in patients with cCHD may drive neurodevelopmental impairments, particularly poorer EF performance. However, this study assessed stress markers and EFs cross-sectionally. Future longitudinal studies are needed to investigate whether early alterations of the physiological stress system in patients with cCHD predict poorer long-term neurodevelopmental outcome. Indeed, cortisol-plus-cortisone concentrations may serve as early biomarkers for neurodevelopmental outcome, and rigorous research is needed to explore this hypothesis.

Future studies should also investigate the mediating effects of brain alterations on the association of stress and EF impairments in patients with cCHD. The limbic structures, such as the hippocampus, amygdala, and prefrontal cortex, are especially vulnerable to stress and increased glucocorticoid release.^56^ Studies in adolescents with cCHD have shown that alterations in these brain regions are associated with impaired working memory, a core domain of EF.^57,58^ HPA dysfunction and associated increased cortisol levels may mediate these associations.

Of note, we computed an age-adjusted EF summary score based on an extensive neuropsychological test battery. The simple, explicit and replicable standardization procedure which was applied in this study did work well for our purpose, and could also be easily used in other studies. We used the data of the controls for age-adjusted standardization but not normative data because of substantial qualitative differences in the available norms for these tests. While this approach may be limited by the differences in parental education of patients and controls, our summary score is not biased by different norms for different tests (eg, regional origin and language of norm samples) and we performed adequate age-adjustment. This is particularly important considering the steep developmental trajectory of EF performance which parallels the maturation of brain network at this age.^59^ At last, using a summary score helps reduce multiple testing and type 1 error in studies. Considering that in patients with CHD, all EF domains seem to be equally affected,^12^ the use of a summary score seems valid.

### Resilience

The current study showed similar self-reported resilience in early adolescents with cCHD and in healthy peers and no significant association with stress markers and EF. These results contradict published findings. Köble and colleagues have shown that adolescents with cCHD score significantly lower than controls in a self-reported resilience questionnaire.^25^ This study used the 11-item version (RS-11) of the same questionnaire that was used in our study (RS-13). Another study found higher resilience in adolescents with cCHD relative to controls assessed with the Connor-Davison Resilience Scale, which assesses similar personality traits as the RS-13.^60^ Considering these different results of 3 studies that used similar methodology (ie, age range, well-powered samples and validated self-reported resilience questionnaires), we assume that variability in resilience among patients with cCHD is very high. Future studies are needed to further elaborate resilience in cCHD and should particularly investigate predictors that can explain the large variability in outcome.

A study in healthy young adults showed that higher scores in a resilience questionnaire were associated with lower hair cortisol.^61^ Some studies also suggest that traits associated with resilience such as positive reappraisal and coping are directly dependent on EFs, indicating an interconnection between resilience and EF.^21,22,62^ Different interpretations and measurements of resilience render comparison across studies difficult. In this study, we used a standardized questionnaire, which assessed the personality traits of acceptance of self and life and personal competences.^35^ However, King and colleagues suggested that resilience should be measured not only with self-report questionnaires but also with observational behavioral measures such as measuring how children cope with stressful tasks to better understand this complex construct.^63,64^ This is relevant as challenges in EF, which are common in cCHD, may hinder introspection. Introspection is crucial for completing self-reported questionnaire. Consequently, studies that incorporate diverse information sources to assess resilience are likely more reliable in the context of EF difficulties.

### Clinical Implications

Our results indicate high stress markers and an association with EF impairments in adolescents with cCHD. Interventions to reduce early life stress during hospitalization may reduce long-term alterations in the HPA axis and contribute to better neurodevelopmental outcomes in these patients. These interventions include reduced parent–infant separation, kangaroo care,^65,66^ promoted breastfeeding,^67^ music therapy,^68^ and/or trauma-informed care in the ICU.^69,70^ Promoting parental and child mental health throughout development may also reduce child stress and be an important target for interventions.^71^ Further studies with longitudinal designs and well-powered sample sizes should investigate these associations and may consider testing the usability of hair cortisol plus cortisone as a predictive biomarker for EFs in patients with cCHD.

### Limitations

The limitations of our study need to be considered. This study is cross-sectional, and thus, no conclusions can be drawn about causality. Although hair cortisol-plus-cortisone levels are well-established markers for HPA axis activity and long-term stress,^8,9^ some confounders such as frequency of hair washing and hair color can influence the cortisol-plus-cortisone concentration.^72^ We excluded hair samples with dyed hair.

We analyzed physiological stress markers with cortisol-plus-cortisone levels, but we did not assess participants’ perceived psychological stress. Cortisol levels have been shown not to correlate well with perceived stress measured with standardized psychological questionnaires.^9,73^ Importantly, we found that differences in stress markers between patients and controls were not associated with recently experienced life events. Nevertheless, we acknowledge that the questionnaire used to assess life events may not capture the full range of stressful experiences. Thus, we cannot exclude that stress markers may be influenced by recently experienced stress.

Additionally, measuring resilience with the RS-13 may not have fully captured participants’ resilience, which has been described as a multifactorial and dynamic construct.^74^ More research is needed to identify more comprehensive ways of measuring this concept.

Additionally, while patients were recruited prospectively, controls were recruited cross-sectionally. Different recruitment approaches may limit the group comparison. Also, this was a single-center study with a relatively homogenous sample of controls who performed well on cognitive tests (mean IQ = 110.8), had high average parental education and more financial resources than patients. This may have led to less variability and therefore less pronounced effects in the association between stress markers and EFs within the control sample. Also, differences in childhood affluence, advantage, and opportunity may exist between the 2 groups and this limits the generalizability of our results. Nevertheless, all analyses were controlled for parental education and group differences remained significant.

## Conclusions

This case-control study is the first, to our knowledge, to provide evidence that early adolescents with cCHD, who underwent infant open heart surgery, showed higher stress markers and lower EF performance than controls. These findings align with previous studies in infants and young children with cCHD showing altered stress markers in this population and underline the chronic burden of cCHD. The group interaction we found shows an association between higher stress markers and lower EF performance in the cCHD group, indicating that chronically high stress markers may play a role in the development of EF impairments in early adolescents with cCHD. Longitudinal studies are needed to better understand the neurobiological mechanisms and timing of alterations in the stress system and its role in neurodevelopmental outcomes in patients with cCHD.

## Notes

Supplement 1. **eFigure.** Recruitment Flow Chart **eText 1.** EF Summary Score **eTable 1.** Statistical Estimates for Each EF Test **eTable 2.** Weights for EF Domains **eTable 3.** Descriptive Statistical Estimates for EF Domains and Global Score in Healthy Controls **eText 2. **Comparison of Participants vs Non-Participants **eTable 4.** Comparison of Patient Data to Healthy Controls and Norms for Each EF Test **eTable 5.** Group Difference Between Patients With Uni- or Biventricular CHD **eTable 6.** Association Between Stress Markers and Clinical Variables **eTable 7.** Associations Between Stress Markers and EFs **eTable 8.** Association Between Resilience, Stress Markers and EFs

Supplement 2.
Data Sharing Statement
