# Supplementary material for: Stress Markers, Executive Functioning, and Resilience Among Early Adolescents With Complex Congenital Heart Disease
Source: JAMA Netw Open. 2024 Feb 9;7(2):e2355373. doi: 10.1001/jamanetworkopen.2023.55373 (PMC10858402; doi:10.1001/jamanetworkopen.2023.55373)
Supplement: Supplement 1. — eFigure. Recruitment Flow Chart eText 1. EF Summary Score eTable 1. Statistical Estimates for Each EF Test eTable 2. Weights for EF Domains eTable 3. Descriptive Statistical Estimates for EF Domains and Global Score in Healthy Controls eText 2. Comparison of Participants vs Non-Participants eTable 4. Comparison of Patient Data to Healthy Controls and Norms for Each EF Test eTable 5. Group Difference Between Patients With Uni- or Biventricular CHD eTable 6. Association Between Stress Markers and Clinical Variables eTable 7. Associations Between Stress Markers and EFs eTable 8. Association Between Resilience, Stress Markers and EFs [file jamanetwopen-e2355373-s001.pdf]

Supplemental Online Content

von Werdt L, Binz TM, O'Gorman RT, et al. Stress markers, executive functioning, and resilience among early adolescents with complex congenital heart disease. *JAMA Netw Open.* 2024;7(2):e2355373. doi:10.1001/jamanetworkopen.2023.55373

eFigure. Recruitment Flow Chart

eText 1. EF Summary Score

eTable 1. Statistical Estimates for Each EF Test

eTable 2. Weights for EF Domains

eTable 3. Descriptive Statistical Estimates for EF Domains and Global Score in Healthy Controls

eText 2. Comparison of Participants vs Non-Participants

eTable 4. Comparison of Patient Data to Healthy Controls and Norms for Each EF Test

eTable 5. Group Difference Between Patients With Uni- or Biventricular CHD.

eTable 6. Association Between Stress Markers and Clinical Variables

eTable 7. Associations Between Stress Markers and EFs

eTable 8. Association Between Resilience, Stress Markers and EFs

This supplemental material has been provided by the authors to give readers additional information about their work.

© 2024 von Werdt L et al. *JAMA Network Open.*

eFigure: *Recruitment Flow Chart*

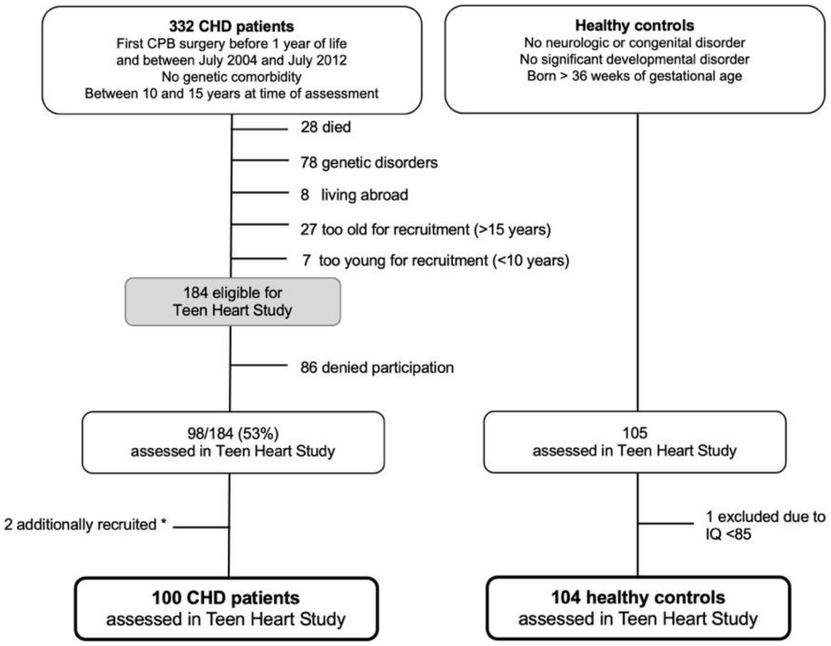

*Note: Patients were recruited from two longitudinal cohort studies conducted at the University Children's Hospital Zurich. a) Spillmann et al., Pediatric Research, 2021. b) von Rhein et al., JPeds, 2015. * 2 patients were additionally recruited from the developmental outpatient clinic with the same inclusion criteria but families did not originally participate in the cohort study. CHD = congenital heart disease. CPB = cardiopulmonary bypass.*

© 2024 von Werdt L et al. *JAMA Network Open.*

***eText 1**: EF Summary Score*

We standardized the raw scores of each test using data from the healthy controls, avoiding the use of normative scores with substantial qualitative differences in the available test norms. The norms for the tests used in this study were published between 2000 and 2012, indicating that some norms were not up-to-date (e.g., RWT from 2000, D-KEFS from 2001). In addition, there were relevant differences in age-adjustment intervals between norms (e.g., WISC-IV with 4-month intervals, RWT with 3-year intervals). Furthermore, age-adjusted norms were unavailable for some tests, such as Corsi Block and TAP Flexibility. The regional origins of the norms also varied, with the WISC-IV from German-speaking countries, RWT and TAP from Germany, and D-KEFS from the US. Therefore, to maintain consistency across tests and ensure adequate age adjustment when creating a summary score for EF, we used the healthy control data for standardization.

Test scores had first to be standardized as standard deviation scores (SDS) so that they could be combined together. Due to a positive linear association between test performance and age in the control sample, this standardization was adjusted for age at assessment. For each test, least square regression was conducted with age as the predictor and the natural logarithm test measurement as the response, to get approximately normally distributed residuals, so that we could estimate an intercept α, a slope β and a residual standard deviation σ. To avoid the undue influence of a few outliers, a robust estimate of σ was obtained by dividing the difference between the 97.5% and 2.5% empirical quantiles of the residuals by 3.92 (=2x1.96). Estimates *for each test are provided in eTable 1.* An age-adjusted standard deviation score (SDS) for a given test was obtained as follows:

$$SDS = SIGN(\beta)*(LN(TEST\ MEASUREMENT) - \alpha - \beta *AGE)/ \sigma$$

In this formula, the multiplication by the sign of beta was done to ensure that better performance was associated with higher SDS for each test. Scores for the five EF domains were calculated by averaging the SDS of the corresponding tests, using importance weights provided in *eTable 2*. The averages were approximately normally distributed with approximately zero mean. In order to get a standard deviation of approximately one, and thus to get SDS for components, these averages were then divided by a standardization constant *tau* (eTable 2). Using basic rules of variance calculation, values of *tau* were determined based on the weights and Spearman's *r* correlations among the SDS of the tests hence averaged. Finally, SDS for the EF summary score were obtained by averaging the SDS of the five EF domains, and by dividing this average by another value of *tau* (*eTable 2*). Note that to maintain robustness of the EF summary score, SDS of the tests and EF domains were limited to ±4 before being averaged. Descriptive statistics of SDS in the healthy controls are provided in *eTable 3.*

© 2024 von Werdt L et al. *JAMA Network Open.*

eTable 1:*Statistical estimates for each EF test*

Test	α	β	σ
Digit Span (WISC-IV)	2.483	0.0290	0.195
Letter-number sequencing (WISC-IV)	2.831	0.0150	0.102
Corsi Block Tapping Test (Corsi)	2.598	0.0292	0.173
Interference, Colour Word Interference Task (D-KEFS)	5.393	-0.1040	0.236
Go/NoGo (TAP)	6.772	-0.0579	0.199
Letter-Number-Switching, Trail Making Task (D-KEFS)	5.331	-0.0886	0.302
TAP Flexibility (TAP)	7.927	-0.0991	0.179
S-words (RWT)	1.973	0.0633	0.281
Animals (RWT)	2.729	0.0561	0.238
Filled-dots-only, Design Fluency Test (D-KEFS)	2.079	0.0120	0.287
Tower Task (D-KEFS)	2.580	0.0215	0.176

© 2024 von Werdt L et al. *JAMA Network Open.*

***eTable 2**: Weights for EF domains*

EF domains	Weights	*tau*
Working memory	0.25*Digit span+0.25*letter number sequencing +0.5*corsi block	0.851
Inhibition	0.5*Go/NoGo+0.5*Color word interference	0.739
Flexibility	0.5*Trail Making Test+0.5*TAP Flexibility	0.858
Fluency	0.25*s-words +0.25*animals +0.5* design fluency	0.751
Planning	1* tower task	1.000
EF summary score	0.2* Working memory +0.2* Inhibition +0.2* Flexibility +0.2* Fluency +0.2* Planning	0.696

eTable 3: Descriptive statistical estimates for EF domains and global score in healthy controls.

Component	Mean	SD	Skewness	Median	IQR/1.35	Yule
Working memory	0.00	0.94	-0.15	0.04	0.96	-0.08
Inhibition	-0.01	1.09	-0.18	0.06	1.05	-0.10
Flexibility	0.00	0.98	-0.19	0.18	1.12	-0.33
Fluency	0.03	0.98	-0.50	0.06	1.02	0.04
Planning	0.00	1.03	-0.55	0.07	0.87	0.05
Global EF score	0.01	0.96	-0.46	0.20	0.92	-0.22

Note. Ideally, estimates should have mean or median equal to 0, SD/IQR equal to 1 and skewness or Yule coefficient equal to 0, which was approximately the case.

eText 2: Comparison of participants vs. non-participants

Patients who participated in this study did not significantly differ from patients who were eligible but rejected to participate in respect to sex (X^2=0.34,*P*=0.561), time on ICU (*W*=4796,*P*=0.106), having a univentricular/biventricular cCHD (X^2=0.24,*P*=0.621). There was a trend of higher parental education in participants vs. non-participants (*W*=4527,*P*=0.051).

© 2024 von Werdt L et al. *JAMA Network Open.*

eTable 4: *Comparison of patient data to healthy controls and norms for each EF test*

	CHD	*Controls*	*P CHD vs. controls* [a]	*P CHD vs. norm* [b]
**Working memory**				
Digit Span[c] (scaled score, 10±3)	9.1±3.1	11.5±2.9	<0.001	0.02
Letter-number sequencing[c] (scaled score, 10±3)	9.6±3.1	11.9±2.3	<0.001	0.26
Corsi Block Tapping Test (raw score)	14.9±3.1	16.7±2.8	<0.001	-
**Inhibition**				
Colour Word Interference Task[d] (scaled score, 10±3)	9.3±3.0	11.3±2.5	<.001	0.04
Go/NoGo [e] (*t* score, 50±10)	52.1±10.7	52.2±10.3	0.96	0.08
**Cognitive flexibility**				
Trail Making Task[d] (scaled score, 10±3)	9.5±3.4	11.4±1.9	<0.001	0.15
Flexibility (raw score)	939.8±306.1	784.1±183.8	<0.001	-
**Fluency**				
S-words, verbal fluency[f] (percentiles)	36.9±29.0	45.8±26.1	<0.001	<0.001
Animals, verbal fluency[f] (percentiles)	37.2±28.4	55.0±30.1	<0.001	<0.001
Design Fluency Test[d] (scaled score, 10±3)	10.4±2.9	11.4±2.6	0.03	0.22
**Planning**				
Tower Task[d] (scaled score, 10±3)	10.1±2.3	11.0±2.1	0.01	0.85

Note: Test values = Mean±SD. [a]two-sampled t test. [b]one-sampled t-tests. ***Information on normative data:*** *[c]* ***WISC-IV****: German-speaking norms (Austria, Germany, Switzerland) published in 2007, adjusted by age with 4-month intervals, sample size 10-15 years = 900; [d]* ***D-KEFS****: US norms published in 2001, adjusted by age with 1-year intervals, sample size 10-15 years = 550; [e]* ***TAP****: German norms published in 2012, adjusted by age adjusted by age with 1-year intervals, sample size 10-15 years = 285; [f]* ***RWT****, German norms published in 2000, adjusted by age with 3-year intervals, sample size 10-15 years = 129; For the Corsi Block Tapping Test and the Flexibility no age-appropriate normative data was available.*

© 2024 von Werdt L et al. *JAMA Network Open.*

eTable 5: *Group difference between patients with uni- or biventricular CHD.*

**Outcome**	**Predictors**	***ß(CI-95)*** *standardized*	***B(CI-95)*** *unstandardized*	***P*** *Uncorrected*	***P*** *FDR-corrected*	***R^2*** *adjusted*	***P*** *Model fit*
Stress markers	Type of CHD[a]	-0.01(-0.23 to 0.21)	-0.01(-0.29 to 0.27)	0.93	0.98	-0.01	0.48
	Sex	0.19(-0.03 to 0.41)	0.22(-0.05 to 0.49)	0.11			
	Age	-0.06(-0.29 to 0.18)	-0.02(-0.13 to 0.08)	0.65			
	Parental education	-0.11(-0.34 to 0.11)	-0.03(-0.09 to 0.03)	0.32			
Executive functions	Type of CHD[a]	0.09(-0.10 to 0.29)	0.30(-0.34 to 0.94)	0.35	0.98	0.05	0.06
	Sex	-0.07(-0.27 to 0.13)	-0.19(-0.77 to 0.39)	0.52			
	Age	0.07(-0.13 to 0.28)	0.08(-0.15 to 0.31)	0.48			
	Parental education	0.29(0.10 to 0.48)	0.17(0.05 to 0.29)	0.01			
Resilience	Type of CHD[a]	-0.00(-0.22 to 0.22)	-0.06(-5.03 to 4.91)	0.98	0.98	-0.00	0.45
	Sex	0.13(-0.09 to 0.35)	2.58(-1.85 to 7.00)	0.25			
	Age	0.07(-0.15 to 0.29)	0.54(-1.24 to 2.31)	0.55			
	Parental education	0.14(-0.08 to 0.36)	0.57(-0.34 to 1.47)	0.21			

Note. [a]Reference group: univentricular CHD. CHD = congenital heart disease

© 2024 von Werdt L et al. *JAMA Network Open.*

***eTable 6**: Association between stress markers and clinical variables.*

Note. CPB = cardiopulmonary bypass. ICU = intensive care unit. All clinical variables (except for number of CPB surgeries) were assessed at the time of the first CPB surgery.

**Predictors**	***β(CI-95)*** *standardized*	***B(CI-95)*** *unstandardized*	***P*** *Uncorrected*	***P*** *FDR-corrected*	***R²*** *adjusted*	***P*** *Model fit*
Number of CPB surgeries	-0.02(-0.24 to 0.21)	-0.01(0.11 to 0.52)	0.89	0.97	-0.00	0.48
Sex	0.19(-0.03 to 0.42)	0.22(0.13 to 0.50)	0.11			
Age	-0.05(-0.28 to 0.19)	-0.02(-0.08 to 0.06)	0.66			
Parental education	-0.11(-0.34 to 0.11)	-0.03(-0.05 to 0.03)	0.32			
Time on CPB	-0.01(-0.23 to 0.22)	0.00(-0.00 to 0.00)	0.97	0.97	-0.00	0.48
Sex	0.19(-0.03 to 0.42)	0.22(-0.05 to 0.49)	0.11			
Age	-0.06(-0.29 to 0.18)	-0.02(-0.13 to 0.08)	0.65			
Parental education	-0.12(-0.34 to 0.11)	-0.03(-0.09 to 0.03)	0.32			
Age at CPB surgery	0.01(-0.22 to 0.25)	0.00(-0.05 to 0.05)	0.91	0.97	-0.00	0.48
Sex	0.19(0.04 to 0.42)	0.22(-0.05 to 0.49)	0.11			
Age	-0.06(-0.30 to 0.18)	-0.03(-0.14 to 0.09)	0.64			
Parental education	-0.11(-0.34 to 0.11)	-0.03(-0.09 to 0.03)	0.34			
Time in ICU	0.17(-0.04 to 0.39)	0.13(-0.04 to 0.30)	0.13	0.52	0.03	0.23
Sex	0.18(-0.04 to 0.40)	0.21(-0.06 to 0.47)	0.13			
Age	-0.03(-0.26 to 0.20)	-0.01(-0.12 to 0.09)	0.80			
Parental education	-0.12(-0.33 to 0.10)	-0.03(-0.09 to 0.03)	0.31			

© 2024 von Werdt L et al. *JAMA Network Open.*

***eTable 7**: Associations between EF and stress markers*

**Model** (Outcome: EF)	**Predictors**	***β(CI-95)*** *standardized*	***B(CI-95)*** *unstandardized*	***P***	***R^2*** *adjusted*	***P*** *Model fit*
Whole sample	Stress markers	-0.06(-0.21 to 0.08)	-0.13(-0.43 to 0.16)	0.38	0.18	<0.001
	Group	-0.32(-0.47 to -0.17)	-0.78(-1.16 to -0.40)	<0.001		
	Sex	-0.03(-0.17 to 0.11)	-0.07(-0.42 to 0.27)	0.68		
	Age	-0.004(-0.14 to 0.13)	-0.00(-0.13 to 0.12)	0.95		
	Parental education	0.19(0.05 to 0.34)	0.09(0.02 to 0.17)	0.01		
Whole sample with interaction effect	Stress markers	0.09(-0.09 to 0.27)	0.18(-0.20 to 0.56)	0.35	0.21	<0.001
	Group	0.24(-0.22 to 0.71)	0.59(-0.55 to 0.73)	0.31		
	Sex	-0.04(-0.18 to 0.10)	-0.10(-0.44 to 0.25)	0.57		
	Age	-0.002(-0.14 to 0.13)	-0.00(-0.13 to 0.12)	0.98		
	Parental education	0.18(0.04 to 0.32)	0.09(0.02 to 0.16)	0.02		
	Interaction	-0.65(-1.15 to -0.15)	-0.72(-1.29 to -0.15)	0.01		
Patients only	Stress markers	-0.21(-0.43 to -0.003)	-0.49(-1.00 to 0.02)	0.06		
	Sex	-0.12(-0.34 to 0.11)	-0.30(-0.91 to 0.30)	0.32		
	Age	0.01(-0.21 to 0.23)	0.01(-0.22 to 0.24)	0.93		
	Parental education	0.22(0.01 to 0.43)	0.13(0.00 to 0.25)	0.05		
Controls only	Stress markers	0.09(-0.11 to 0.3)	0.15(-0.19 to 0.49)	0.38		
	Sex	0.03(-0.17 to 0.24)	0.06(-0.33 to 0.46)	0.75		
	Age	0.02(-0.18 to 0.22)	0.01(-0.13 to 0.15)	0.87		
	Parental education	0.14(-0.06 to 0.33)	0.06(-0.03 to 0.14)	0.19		

Note: Reference for group comparison: controls. Reference for sex comparison: females. CHD = congenital heart disease, EFs= executive functions. FDR-corrected p-value for Stress markers in patients only p = 0.12 and in controls p = 0.38).

© 2024 von Werdt L et al. *JAMA Network Open.*

***eTable 8**: Associations between resilience, stress markers, and EFs.*

**Outcome**	**Predictors**	***β(CI-95)*** ***standardized***	***B(CI-95)*** ***unstandardized***	***P* value**	***R^2*** ***adjusted***	***P*** ***Model fit***
Stress markers ~ Resilience	Resilience	-0.14(-0.29 to 0.008)	-0.01(-0.02 to 0.00)	0.07	0.11	<0.001
	Group	0.27(0.11 to 0.43)	0.33(0.12to 0.53)	0.002		
	Sex	0.28(0.13 to 0.43)	0.34(0.15 to 0.53)	<0.001		
	Age	-0.02(-0.17 to 0.14)	-0.01(-0.08 to 0.06)	0.82		
	Parental education	0.03(-0.13 to 0.19)	-0.01(-0.03 to 0.05)	0.75		
EFs ~ Resilience	Resilience	0.01(-0.13 to 0.14)	0.001(-0.02 to 0.02)	0.93	0.23	<0.001
	Group	-0.32(-0.46 to -0.18)	-0.81(-1.19 to -0.43)	<0.001		
	Sex	0.04(-0.1 to 0.17)	0.10(-0.26 to 0.45)	0.60		
	Age	0.001(-0.13 to 0.14)	0.001(-0.13 to 0.14)	0.99		
	Parental education	0.27(0.14 to 0.41)	0.14(0.07 to 0.22)	<0.001		

Note: Reference for group comparison: controls. Reference for sex comparison: females. CHD = congenital heart disease, EFs= executive functions.

© 2024 von Werdt L et al. *JAMA Network Open.*
