# Supplementary material for: Stress Markers, Executive Functioning, and Resilience Among Early Adolescents With Complex Congenital Heart Disease
Source: JAMA Netw Open. 2024 Feb 9;7(2):e2355373. doi: 10.1001/jamanetworkopen.2023.55373 (PMC10858402; doi:10.1001/jamanetworkopen.2023.55373)
Supplement: Supplement 2. — Data Sharing Statement [file jamanetwopen-e2355373-s002.pdf]

Data Sharing Statement

von Werdt. Stress Markers, Executive Functioning, and Resilience Among Early Adolescents With Complex Congenital Heart Disease. *JAMA Netw Open*. Published February 09, 2024. doi:10.1001/jamanetworkopen.2023.55373

Data
Data available: Yes
Data types: Deidentified participant data
How to access data: request sent to melanie.ehrler@kispi.uzh.ch (corresponding author)
When available: With publication

Supporting Documents
Document types: None

Additional Information
Who can access the data: for research purpose only, after ethical approval and signed data sharing agreement
Types of analyses: for research purpose only
Mechanisms of data availability: after approval of proposal, ethical approval and signed data sharing agreement
Any additional restrictions: research purpose only
